# Diversity and structure of riparian forests in an industrial and urban hub of the Amazon

**DOI:** 10.1007/s10661-025-14970-y

**Published:** 2026-01-08

**Authors:** Julia Isabella de Matos Rodrigues, Walmer Bruno Rocha Martins, Matheus de Lima Guedes, Francisco de Assis Oliveira, Gracialda Costa Ferreira

**Affiliations:** 1 Federal Rural University of Amazonia, Av. Presidente Tancredo Neves, 2501, Belém, Pará 66077-530 Brazil; 2 Federal Rural University of Amazonia, Pau Amarelo Street, W/N, Vila Nova, Capitão Poço, PA 68650-000 Brazil

**Keywords:** Anthropization, Floristic composition, Biomass stock, Floristic diversity

## Abstract

Barcarena stands out as an industrial hub in the Eastern Amazon, hosting a range of activities, including industrial operations and urban expansion, that exert intense pressure on riparian forests. These forests play a strategic role in providing ecosystem services such as hydric regulation and product provision. Understanding the structure and floristic diversity of these areas therefore provides insights into environmental management, particularly for environmental licensing and the restoration of degraded areas. This study aimed to assess the diversity of riparian vegetation in areas within the industrial complex of the Eastern Amazon. Riparian forest formations were classified based on their distribution along the three main water systems located in the municipality of Barcarena: the Murucupi River, Marajó Bay, and Barcarena River. In each area, four plots measuring 25 × 100 m (2500 m^2^) were established, and a floristic inventory was conducted, including all tree and shrub individuals with a diameter ≥ 3.82 cm. A total of 1385 individuals were recorded, distributed across 53 botanical families and 209 species. The area associated with Marajó Bay exhibited a lower individual density than that of the Barcarena River. When standardized by the number of individuals, the species richness of Murucupi River forests was lower than that of the other sites. Nonetheless, similarities in floristic composition were observed among the areas. These results indicated that the conservation of riparian ecosystems in multi-use landscapes requires attention not only to forest structure but also to ecological integrity, as changes in species composition may precede deeper functional losses.

## Introduction

Riparian forests are composed of plant species that grow in terrestrial ecosystems under fluvial influence (Zheng et al., 2023) and play crucial ecological and socio-environmental roles. In tropical regions, the high species diversity of these ecosystems sustains the livelihoods of riparian communities (Sanches et al., 2021) and holds global significance as carbon sinks, which are essential for mitigating climate change (Sutfin et al., 2016; Verdonschot & Verdonschot, 2023). However, in recent decades, riparian forests have been increasingly subjected to anthropogenic pressures, primarily driven by urban and industrial expansion, land use conversion, infrastructure development, and the growing demand for natural resources (Sharma & Khanal, 2024). Consequently, such degradation compromises the structure and floristic composition of these ecosystems, leading to the loss of key functions, such as the regulation of erosion and river siltation risks, as well as the reduced provision of food resources for aquatic fauna (Arantes et al., 2018; Silva et al., 2017).

In the Amazon, riparian forests show contrasting ecological patterns, because well-conserved areas typically maintain high floristic diversity and complex structure (Méndez-Toribio et al., 2014), whereas degraded riparian zones exhibit reduced species richness, biotic homogenization, and shifts toward pioneer-dominated communities (Vallejo et al., 2022). Documented impacts include declines in tree biomass, simplification of vertical structure, alteration of regeneration dynamics, and loss of specialist species (Giese et al., 2003; Maracahipes-Santos et al., 2020; Seidler, 2017).

In regions where multiple human activities overlap, it becomes challenging to isolate the effects of a single source of impact on fluvial and riparian ecosystems. In the Amazonian context, the municipality of Barcarena is recognized as an industrial hub, characterized by an intense overlap of land uses and the close proximity of industrial areas, ports, and urbanized zones. Historically, this region has been a focus of environmental concern due to its growing industrial density and hydrological vulnerability, as anthropized areas encompass an extensive network of natural drainage systems. These systems include important ecological corridors and remnants of riparian forests, which are legally protected as Permanent Preservation Areas under Brazilian legislation (Brasil, 2012).

Although the Brazilian Forest Code (Brasil, 2012) establishes protective regulations, several studies have shown that compliance and enforcement in heavily industrialized regions such as Barcarena remain limited, resulting in continued deforestation, pollution events, and illegal occupation of riparian zones (Lopes et al., 2021). Understanding the ecological patterns of these forests is therefore essential for informing local management strategies and improving the effectiveness of existing regulatory instruments.

Anthropogenic activities raise concerns regarding the ecological integrity of riverbanks, as riparian vegetation is highly sensitive to both water quality and soil dynamics. This highlights the need to assess whether proximity gradients to industrial, urban, and recreational complexes lead to structural and compositional changes in riparian plant communities (Silva et al., 2017). Therefore, approaches that consider hydrography as the analytical unit are essential for understanding environmental disturbance gradients and their ecological implications. In this study, we classified the evaluation areas within the municipality of Barcarena based on the main fluvial systems, aiming to more accurately capture the environmental contexts and the specific types of pressures to which local riparian forests are subjected. Accordingly, three hydrographic units with contrasting levels of anthropogenic interference were selected: the Murucupi River, Marajó Bay, and the Barcarena River (Lopes et al., 2021).

This study aimed to assess the structure, diversity, and floristic composition of riparian forests associated with the Murucupi River, Marajó Bay, and Barcarena River in the Eastern Amazon. It answers the following scientific question: What are the differences between fluvial systems in the Barcarena region in terms of the structure and floristic composition of their riparian forests? The hypothesis was that areas closer to urban, industrial, or port zones would exhibit more pronounced negative effects on vegetation.

## Material and methods

### Study site

**Fig. 1:**
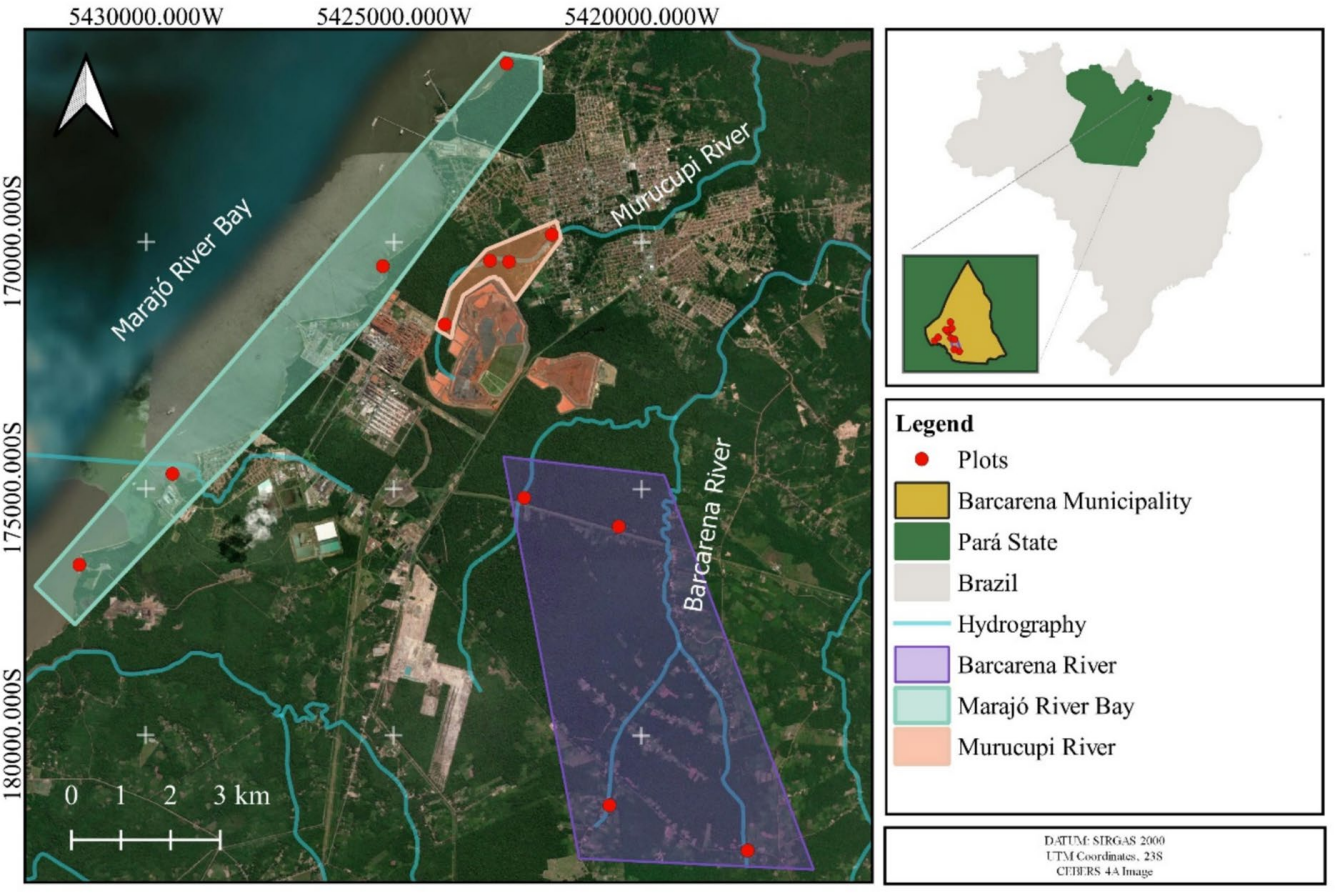
Sampling points in areas of the alumina refinery industrial complex and a reference ecosystem located in the municipality of Barcarena, Brazil, Eastern Amazon

The study was conducted in the municipality of Barcarena, Pará, Eastern Amazon (01°30′21″S; 48°37′33″W; Fig. 1). The climate of the municipality is classified as humid tropical according to Köppen (Alvares et al., 2013). Soils are predominantly Dystrophic Red-Yellow Latossols, Hydromorphic Podzols, and Lateritic Concretionary soils (RADAMBRASIL, 1974). The natural vegetation is classified as Dense Ombrophilous Forest (IBGE 2012); however, it has been largely altered by human occupation, particularly due to industrial and urban expansion in the region (MapBiomas, 2024).

### Delimitation and classification of the study areas

The delimitation of the study areas was based on the distribution of riparian forest formations along three fluvial systems located in the municipality of Barcarena, Pará, Brazil. The selection of these fluvial systems considered the varying levels of anthropogenic interference to which they are subjected, reflecting distinct socio-environmental contexts and forms of land use. Consequently, the adopted categorization aimed to represent a gradient of environmental disturbance, ranging from areas under intense anthropogenic pressure to areas experiencing lower levels of direct impact. Accordingly, the study areas were organized into three categories, corresponding to the Murucupi River, Marajó Bay, and Barcarena River.

The Murucupi River is situated within a highly anthropized landscape, characterized by the presence of industrial areas, densely populated urban centers, and the former municipal landfill of Barcarena. Its course traverses peripheral zones of the urban matrix as well as regions adjacent to industrial hubs, making it potentially affected by multiple sources of diffuse and point-source pollution. The removal of native vegetation along the banks, the discharge of waste, and unplanned occupation contribute to the degradation of the environmental quality of the associated riparian system. For these reasons, the Murucupi River was classified as representative of a high anthropogenic interference scenario.

Marajó Bay, located in a transitional area between urban and estuarine zones, is influenced by a range of continuous and diffuse anthropogenic activities. Among the main pressure factors are the discharge of treated effluents from industrial and residential facilities, boat traffic, port operations, and recreational use of its banks, including river beaches and bathing areas. Although it does not exhibit the concentration of point-source impacts observed in the Murucupi River, Marajó Bay experiences a persistent type of environmental disturbance, the intensity of which is modulated by seasonality and the hydrological dynamics of the system. Due to these characteristics, this area was classified as representative of a moderate and diffuse anthropogenic interference scenario.

The Barcarena River corresponds to a less urbanized portion of the municipality, situated away from major industrial hubs and exhibiting lower human occupancy along its banks. The riparian areas along the Barcarena River maintain greater continuity of forest cover and show less evidence of direct intervention, such as deforestation or waste disposal. Although not entirely free from anthropogenic influence, the Barcarena River retains ecological attributes closer to natural conditions and was used in this study as a reference area for comparing the effects of different levels of disturbance observed in the other categories.

### Data collection

A total of 12 plots, each measuring 25 × 100 m, were systematically established, with four plots in each hydrographic influence area. Within each plot, a floristic inventory was conducted for all tree, shrub, and palm individuals with a diameter at breast height (DBH, measured at 1.30 m above ground level) ≥ 3.82 cm. This DBH limit was adopted to include the naturally slender palms and other small-diameter individuals typical of riparian forests, which would be underrepresented using more conventional thresholds (e.g., 5–10 cm). In addition to measuring DBH using diameter tapes, total height (Ht) was estimated using the Biltmore stick method. When in situ species identification was not possible, standardized botanical samples were collected and compared with reference specimens at the IAN Herbarium of Embrapa Eastern Amazon, located in Belém, Pará. Aboveground biomass (AGB) was estimated using the allometric equation proposed by Chave et al. (2014) (Eq. 1), with Neotropical wood density data obtained from the Global Wood Density Database (Chave et al., 2009) for each species. Total AGB was calculated as the sum of all individuals in each influence area and subsequently converted to megagrams per hectare (Mg ha^−1^).

1 $$\mathrm{AGB}={0.0673\times (\rho {D}^{2}H)}^{0.976}$$

where AGB is the aboveground biomass (kg); *ρ* is the wood specific density (g cm^−3^); *D* is the tree diameter (cm); and *H* is the tree height (m).

### Data analysis

For hypothesis testing, a significant level of 5% was considered, and four plots in each area. Species richness similarity among areas was evaluated using individual-based rarefaction curves via Hill numbers, derived from transformations of the Shannon-Weaver and Simpson indices. These transformations, proposed by Hill (1973), assign weights to species based on their relative frequencies (Shannon-Weaver) or to the more common species (Simpson). Sampling effort was standardized by the number of individuals in each sampled area, using the iNEXT package (Chao et al., 2014; Hsieh et al., 2016). In addition to species richness, floristic-structural dissimilarity was assessed through Non-Metric Multidimensional Scaling (NMDS) with 999 permutations, based on the Bray-Curtis index. This analysis was also conducted at the level of individual sampling plots and visualized through a cluster dendrogram. Stress values were calculated as a measure of the quality of the data representation in reduced-dimensional space, with emphasis on achieving a low stress value (≤ 0.2) to indicate a satisfactory graphical representation. Furthermore, a hierarchical clustering analysis was performed using a presence-absence species matrix, based on the Jaccard similarity index. The resulting clusters were visualized in a heatmap with dendrograms, generated using the ggplot2 (Wickham, 2016) and factoextra (Kassambara & Mundt, 2020) packages.

The data were tested for normality and homogeneity of variance using the Shapiro-Wilk and Levene tests, respectively, both at a 5% significance level. Once the assumptions were met, mean species and individual densities were compared among areas using Tukey’s test (*p* < 0.05) following a significant analysis of variance (ANOVA, *p* < 0.05). For aboveground biomass, the assumptions of normality and homoscedasticity were not met. Therefore, differences among areas were evaluated using a Generalized Linear Model (GLM) with Gamma distribution and log link, which is appropriate for continuous, positive, and right-skewed data. When the effect of area was significant (*p* < 0.05), pairwise comparisons were performed using estimated marginal means with Tukey adjustment. All statistical analyses and graphical representations were performed using R software v.4.3.3 (R Development Core Team, 2025).

## Results

**Fig. 2:**
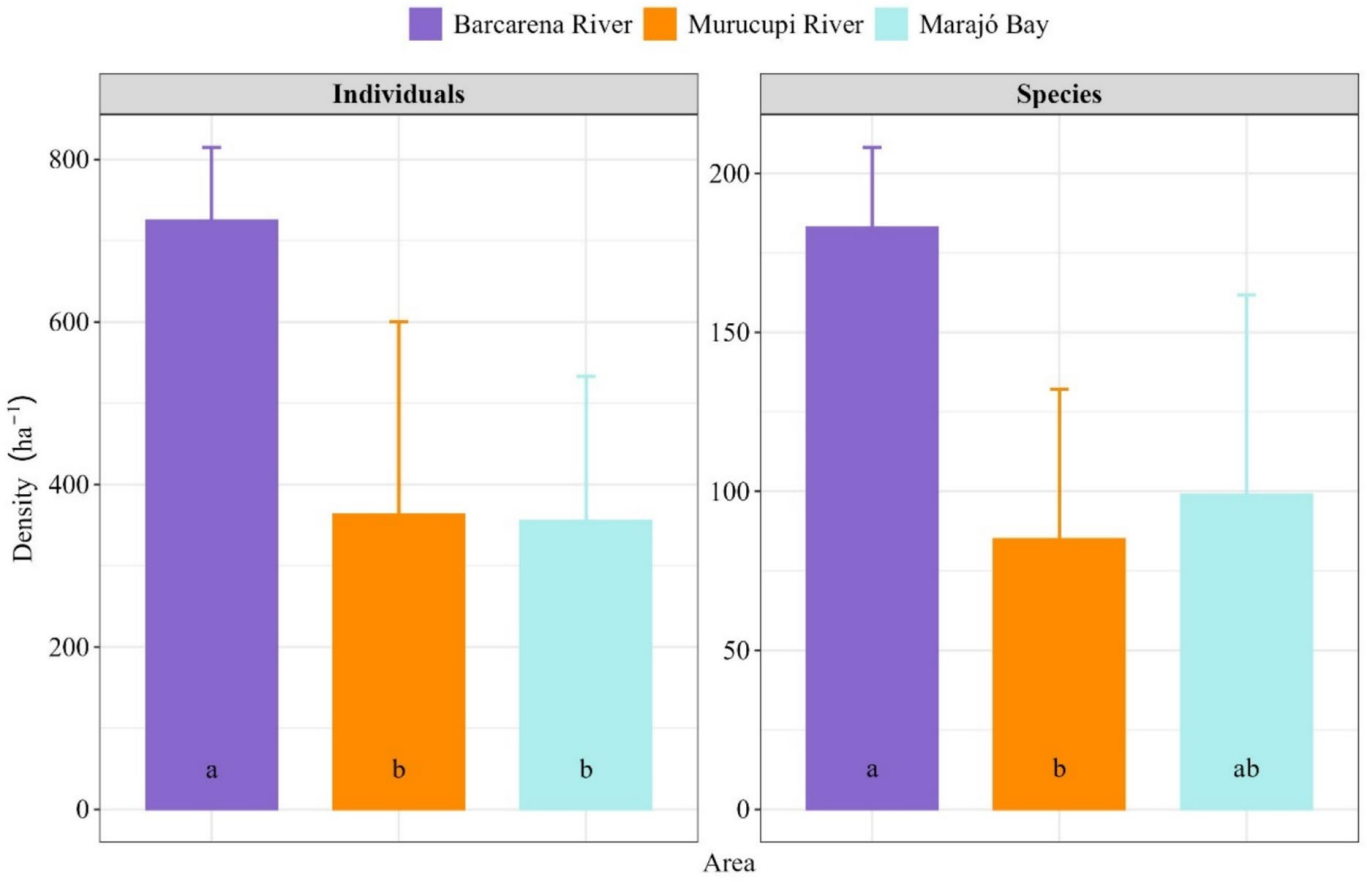
Mean ± standard deviation of individual and species density in riparian forests located within the influence areas of different fluvial systems in the municipality of Barcarena, Eastern Amazon, Brazil

A total of 1385 individuals were inventoried, distributed across 53 botanical families and 209 species. Fabaceae (*n* = 417), Arecaceae (*n* = 185), and Lecythidaceae (*n* = 104) were the families with the highest number of individuals. The families with high species richness were Fabaceae (*n* = 38), Chrysobalanaceae (*n* = 12), and Euphorbiaceae and Lecythidaceae (both with 9 species). The mean individual density ranged from 355.00 ± 89.94 ind. ha^−1^ in forests associated with the Murucupi River to 725.00 ± 89.94 ind. ha^−1^ in those influenced by the Barcarena River (Fig. 2). Both the Murucupi and Marajó Bay forests showed lower values than the Barcarena River (*F*_[2;9]_ = 5.58; *p* > 0.05) for individual density. Regarding species density (Fig. 2), only the Murucupi River forests (85.00 ± 45.15 sp ha^−1^) were significantly lower (*F*_[2;9]_ = 4.96; *p* = 0.04) than those of Barcarena (183.00 ± 25.17 sp ha^−1^).

**Fig. 3:**
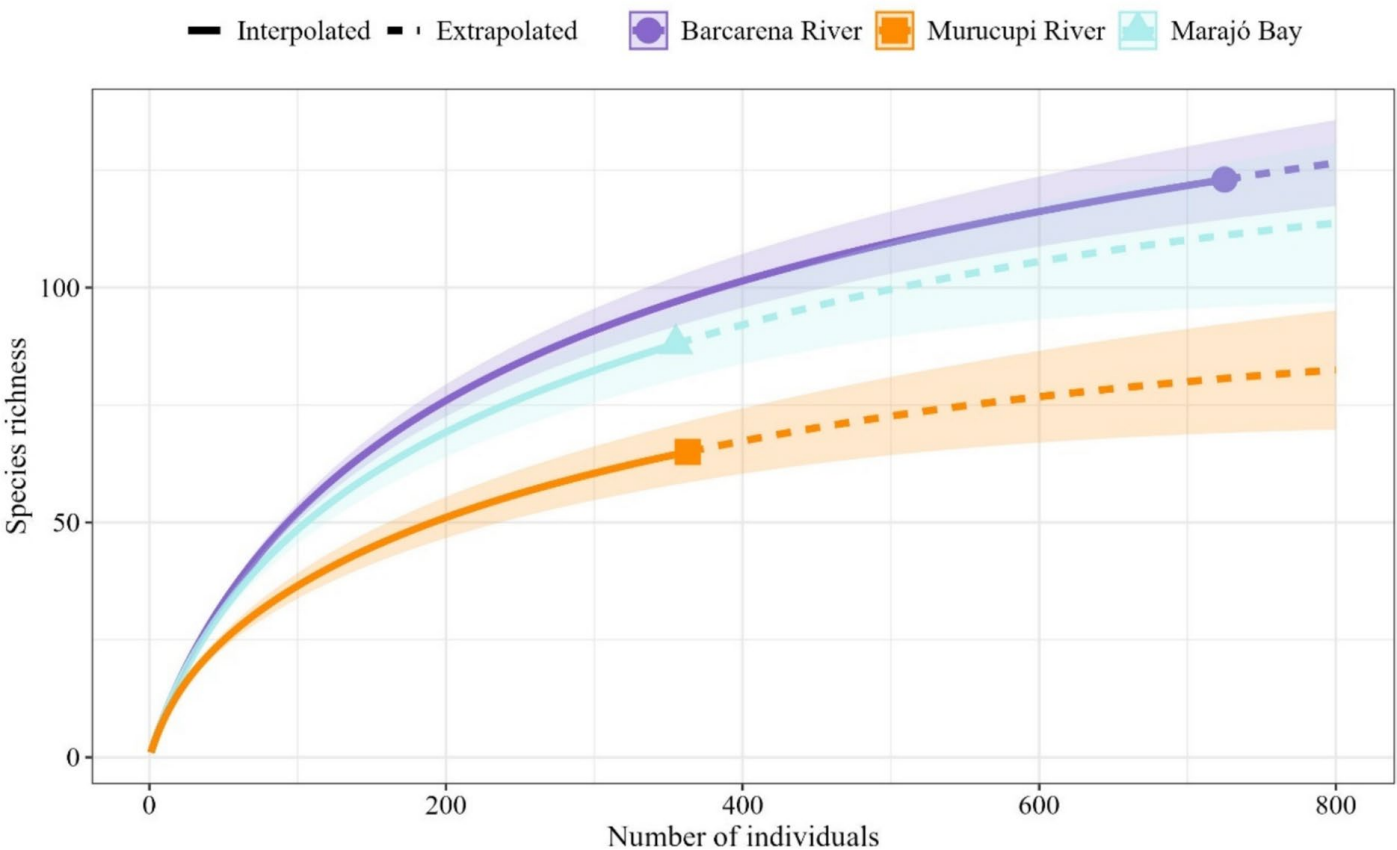
Individual-based rarefaction curves in riparian forests located within the influence areas of different fluvial systems in the municipality of Barcarena, Eastern Amazon, Brazil. Solid lines represent the observed mean species richness, while dashed lines indicate the estimated species richness. Shaded areas correspond to 95% confidence intervals

Rarefaction curves indicated that, when standardizing the number of individuals (*n* = 400), species richness in the Murucupi River (82,46) forests was lower than in the Barcarena River (126,53) and Murucupi River (113,73) (Fig. 3). Extrapolation to 800 individuals showed that the Barcarena River forests had the highest estimated richness (158.95 ± 11.89), followed by Marajó Bay (126.42 ± 21.79), and finally the Murucupi River (91.11 ± 13.48). Regarding diversity, the Barcarena River exhibited the highest Hill numbers based on Shannon (71.88) and Simpson (35.28), indicating a more diverse and equitable community. Intermediate values were observed in Marajó Bay (Hill-Shannon: 63.02; Hill-Simpson: 36.66), whereas the Murucupi River showed the lowest indices (Hill-Shannon: 36.06; Hill-Simpson: 20.50).

**Fig. 4:**
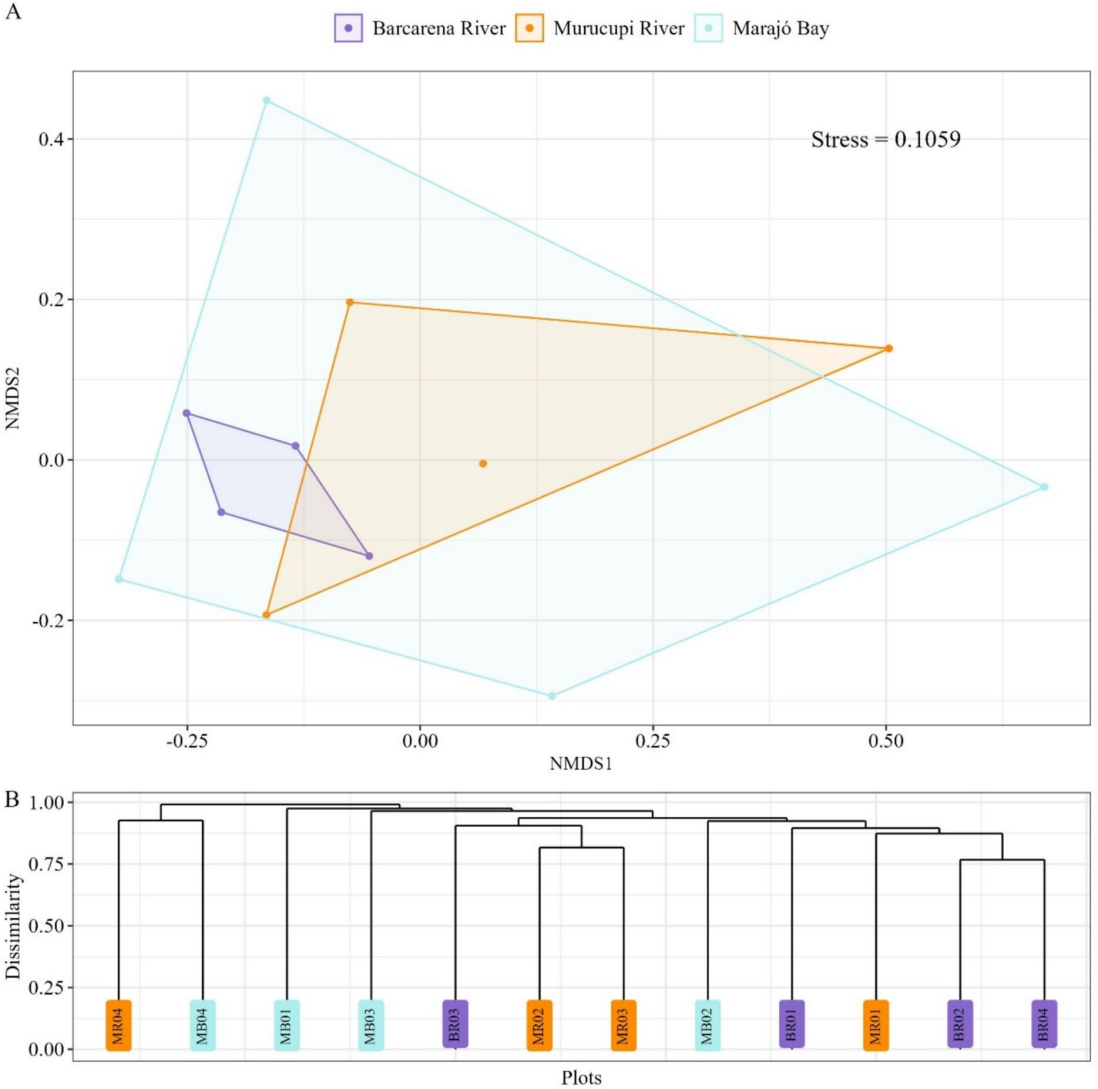
**A** Non-metric multidimensional scaling (NMDS), and **B** cluster dendrogram, both based on the Bray-Curtis dissimilarity index in riparian forests located within the influence areas of different fluvial systems in the municipality of Barcarena, Eastern Amazon, Brazil

NMDS analysis revealed similarities in floristic composition among the areas, with a stress value of 0.11, indicating a good two-dimensional representation of the data (Fig. 4A). In the Barcarena River, points were clustered near the origin (NMDS1 = −0.16 ± 0.08; NMDS2 = −0.03 ± 0.08), showing less overlap with the Murucupi River area. In contrast, Marajó Bay exhibited marked dispersion (NMDS1 = 0.08 ± 0.40; NMDS2 = –0.01 ± 0.32), encompassing the other studied areas and suggesting similarity in floristic composition among them (Fig. 4A). The dendrogram based on the Bray-Curtis distance corroborated the patterns observed in the NMDS analysis (Fig. 4B).

**Fig. 5:**
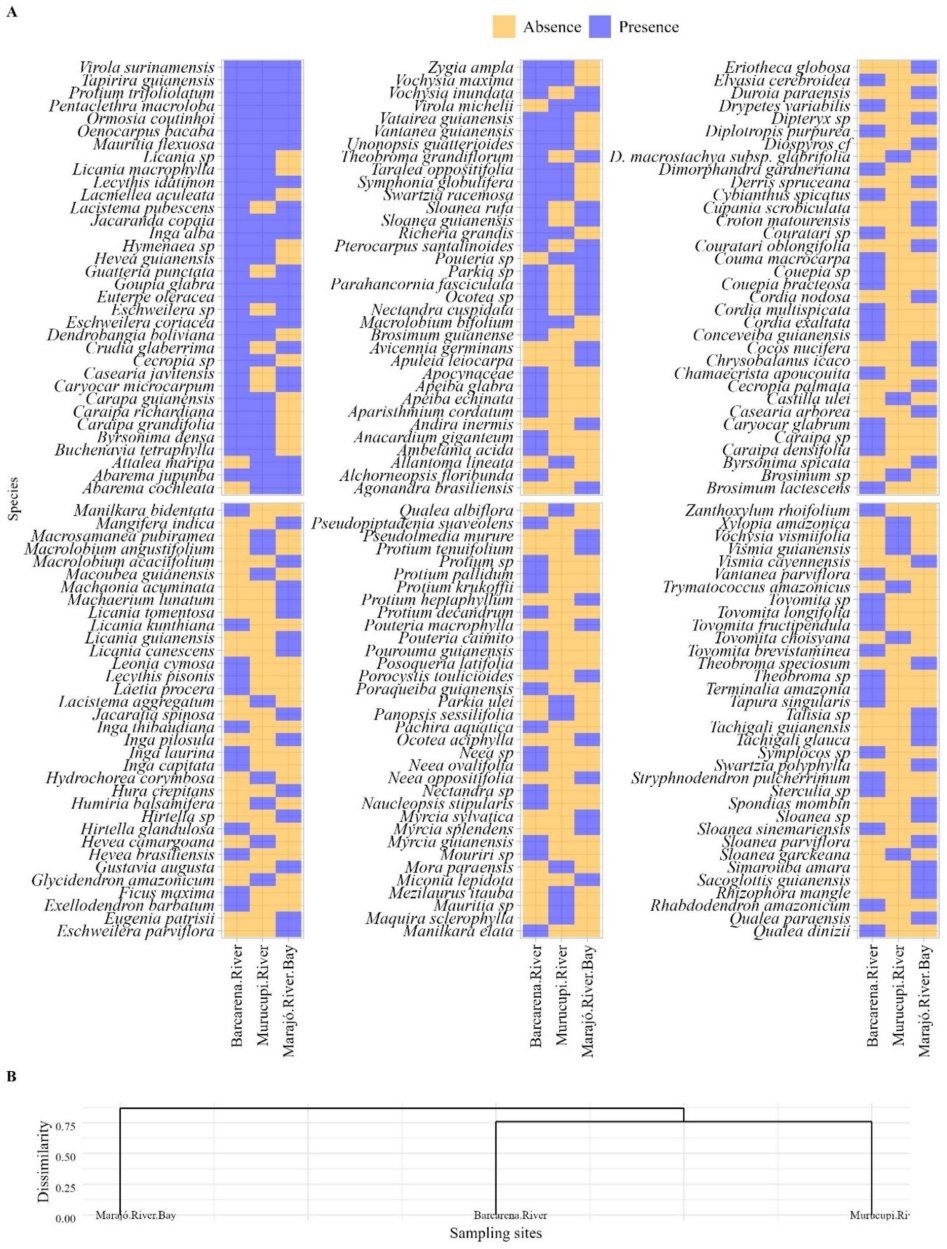
Heatmap (**A**) and cluster dendrogram (**B**) for floristic composition in riparian forests located within the influence areas of different fluvial systems in the municipality of Barcarena, Eastern Amazon, Brazil

Marajó Bay showed less species overlap compared to the other areas, whereas the Murucupi River exhibited species distribution patterns similar to those of the Barcarena River (Fig. 5A). Eleven species were present in all areas: *Virola surinamensis*, *Tapirira guianensis*, *Protium trifoliolatum*, *Pentaclethra macroloba*, *Ormosia coutinhoi*, *Oenocarpus bacaba*, *Mauritia flexuosa*, *Lecythis idatimon, Jacaranda copaia*, *Inga alba*, *Goupia glabra*, *Euterpe oleracea*, *Eschweilera coriacea*, and *Abarema jupunba* (Fig. 5A). In the Barcarena River, 69 unique species were recorded, followed by 25 in the Murucupi River and 16 in Marajó Bay (Fig. 5A). Cluster analysis corroborated these results, forming two main groups: one comprised exclusively of forests from Marajó Bay, and the other including the Barcarena and Murucupi Rivers (Fig. 5B).

Aboveground biomass varied among the areas, ranging from approximately 89 Mg ha^−1^ in Marajó Bay to 142 Mg ha^−1^ in Murucupi River (Table 1). However, the Gamma GLM indicated that these differences were not statistically significant (*p* > 0.05), and no pairwise contrasts showed evidence of areas (Table 1).

**Table 1:** Generalized linear model (gamma, log link) results and estimated marginal means for aboveground biomass in riparian forests located within the influence areas of different fluvial systems in the municipality of Barcarena, Eastern Amazon, Brazil

Parameter	Estimate	Std. error	Mean (Mg ha^−1^)	*p*-value
[Intercept]	4.8066	0.2767	122.32	< 0.001
Murucupi River	0.1495	0.3913	142.04	0.711
Marajó Bay	−0.3133	0.3913	89.42	0.444

## Discussion

This study evaluated the structure and floristic diversity of vegetation in riparian forests located across different fluvial systems in the municipality of Barcarena, Eastern Amazon, which are subject to varying levels of anthropogenic pressure. Comparisons between areas associated with the Murucupi River and Marajó Bay and the area under lower anthropogenic influence (Barcarena River) allowed for an assessment of how these pressures may affect floristic-structural attributes of riparian formations. The results answer the study question, and partially accept the initial hypothesis, because results revealed differences in floristic composition associated with the degree of disturbance, although no significant changes in above ground biomass were observed. These findings contribute to a better understanding of the resilience and vulnerability of these ecosystems under scenarios of intensive land use.

Although the Murucupi River area exhibited lower species richness, the floristic-structural patterns, as well as individual and species densities, were similar to those observed in Marajó Bay. In both areas, the presence of generalist species such as *Mauritia flexuosa* and *Ormosia coutinhoi* likely reflects an adaptive response to potential environmental stresses. Although *M. flexuosa* is typically associated with permanently or seasonally waterlogged soils, its occurrence in sites with varying degrees of moisture indicates a capacity to withstand hydrological fluctuations that are common in anthropized riparian zones (Salvador et al., 2022). *O. coutinhoi*, in turn, is known for persisting across disturbed and relatively conserved habitats, suggesting tolerance to changes in canopy openness, soil conditions, and disturbance regimes (Vale et al., 2014). Furthermore, Marajó Bay presents distinct environmental characteristics, as it is situated in an estuarine environment subject to stronger hydrodynamic influence, which partly explains the differentiated floristic composition and the high dispersion observed in this study. Additionally, this area is under constant anthropogenic pressure, being a port region frequently used for recreational activities.

In the case of aboveground biomass accumulation, the absence of significant differences among the evaluated areas can be attributed to the nature of this structural indicator, which is recognized as being less sensitive to short- or medium-term disturbances due to the slow growth of tree species (Cupertino et al., 2024). Nevertheless, the high data dispersion expressed as the large variability in aboveground biomass values among the sampled areas, especially in Marajó Bay, may indicate long-term effects resulting from environmental degradation. Among the local factors that may have contributed to this pattern is the historical land use, including the occurrence of environmental accidents. A notable example is the 2015 shipwreck at the Vila do Conde port, located in the Pará River, which has been associated with ecological disturbances in Marajó Bay. Consequently, there was a proliferation of opportunistic zooplankton species due to animal decomposition (Pinheiro et al., 2019), which may have influenced the floristic-structural patterns observed in this study.

In Marajó Bay, the occurrence of exclusive species such as *Avicennia germinans* (L.) L. and *Chrysobalanus icaco* L. reflects the presence of waterlogged soils influenced by tidal dynamics, as observed in the field. The presence of *Byrsonima spicata* (Cav.) DC. and *Eriotheca globosa* (Aubl.), species adapted to nutrient-poor soils, aligns with the widely documented low edaphic fertility in the Amazon region. Although not restricted to waterlogged environments, both species are able to tolerate moderately hydromorphic conditions, such as soils with slow drainage or temporary saturation (Amaral et al., 2021; Vieira et al., 2017). Their occurrence therefore reflects the combined influence of low nutrient availability and moisture-retention properties of the local soils, suggesting that the local floristic composition reflects adaptations to nutritionally restrictive conditions. These specific environmental conditions favor adapted and tolerant species in degraded ecosystems, such as *Andira inermis* (W. Wright) DC. (Álvarez et al., 2021; Fanday & Tchobsala, 2024) and *Machaerium lunatum* (L.f.) Ducke (Berger et al., 2006), both members of Fabaceae, which are capable of forming associations with nitrogen-fixing bacteria. Consequently, the establishment of specialized species limits diversity and leads to the predominance of low wood density species, reducing the overall vegetation biomass accumulation, as observed in this study.

According to the rarefaction analysis (Fig. 3), which standardized sampling effort by comparing diversity at an equivalent number of individuals, riparian forests associated with the Barcarena River exhibited a higher density of individuals (Fig. 2). The higher richness observed in this area suggests that, in contexts with lower anthropogenic pressure, riparian forests tend to maintain denser and more diverse plant communities. This finding highlights the potential of these areas as reference sites for ecological restoration efforts, including in degraded zones or areas undergoing revegetation, such as those near urban centers and industrial zones. The high diversity recorded around the control area could serve as a basis for seed banks and the selection of species adapted to local conditions, should reforestation interventions be necessary.

In this context, the use of native species with proven resilience to disturbances should be prioritized in restoration programs, particularly because they are adapted to riparian ecosystems, exhibiting resilience and tolerance to environmental stressors. In this study, *O. coutinhoi* and *M. flexuosa* were highlighted, being present in all evaluated areas. *O. coutinhoi* is a nitrogen-fixing species capable of forming nodules even under nutritional stress (Pons et al., 2007), whereas *M. flexuosa* mitigates oxidative stress caused by soil flooding, ensuring its persistence in these environments (Salvador et al., 2022). Additionally, *T. guianensis* displayed high ecological plasticity, reinforcing its role as a generalist species resistant to environmental disturbances (Ødegaard & Frame, 2007; Santos et al., 2024).

Therefore, although impacts associated with intensive land use did not cause significant changes in aboveground biomass, the results indicated alterations in floristic composition in the more heavily impacted areas. The replacement of sensitive species by generalist and tolerant species, coupled with reduced diversity in certain plots, suggests an initial process of ecological degradation, which could progress to more severe stages if preventive measures are not implemented. The strategic introduction of resilient key species, such as *O. coutinhoi* and *M. flexuosa*, may help maintain ecological functions and mitigate cumulative risks (Pons et al., 2007; Salvador et al., 2022), particularly in Amazonian riparian ecosystems located in regions under strong development pressure, such as the Barcarena industrial hub. In these areas, the synergy between anthropogenic disturbances and extreme events poses a tangible threat to the stability of ecosystem services, highlighting the urgent need for management and conservation strategies informed by local ecological knowledge.

Despite these insights, some limitations of the study should be acknowledged in order to contextualize the strength and scope of the findings. The sampling effort was restricted to four plots per hydrographic unit because access to several areas of Barcarena is limited, as the region contains industrial facilities, private properties, and long-standing socio-environmental conflicts that hinder the establishment of larger sampling designs. In addition, although the study evaluates vegetation structure, this assessment is not exhaustive. Other important structural parameters, such as basal area, diameter class distribution, and the height of tree and shrub strata (Rosenfield et al., 2023), were not included and could be incorporated into future analyses. However, biomass integrates the effects of species composition, wood density, and diameter distribution (Utla et al., 2025), making it sensitive to disturbance gradients commonly observed in riparian environments affected by industrial and urban activities (Araujo et al., 2023).

Even with these constraints, the findings offer a robust basis for understanding how riparian forests respond to varying disturbance levels in industrialized Amazonian landscapes. Therefore, the study provides a valuable comparative overview of floristic composition and vegetation structure in riparian forests exposed to different levels of anthropogenic pressure. The results reinforced the value of Amazonian riparian forests as sensitive indicators of ecological integrity and highlight their relevance for environmental monitoring, management, and restoration planning. Future research should seek to expand the sampling design where access is feasible, incorporate a broader set of structural and functional metrics, and establish long-term monitoring programs to deepen understanding of how disturbance gradients influence the resilience and ecological trajectories of these ecosystems.

## Conclusion

The absence of marked differences in vegetation structure suggests a capacity for maintaining the forest canopy even under the influence of industrial and urban activities in the surrounding areas. However, the floristic composition reflects the influence of multiple historical and environmental factors, including hydrological regime, salinity, and past disturbances, resulting in variations in diversity and the replacement of sensitive species by more generalist and adaptable taxa. These patterns indicate that conservation of riparian ecosystems in areas with overlapping human activities requires attention not only to forest structure but also to ecological integrity, as changes in composition may precede deeper functional losses. Therefore, the maintenance and enhancement of environmental management measures are essential to ensure the resilience and functionality of these strategic ecosystems in the Eastern Amazon.

## Data Availability

No datasets were generated or analysed during the current study.

## Declarations

All authors have read, understood, and have complied as applicable with the statement on “Ethical responsibilities of Authors” as found in the Instructions for Authors.
